# Involvement of µ-Opioid Receptor in Methamphetamine-Induced Behavioral Sensitization

**DOI:** 10.2174/157015911795016949

**Published:** 2011-03

**Authors:** Lu-Tai Tien, Ing-Kang Ho

**Affiliations:** 1 School of Medicine, Fu-Jen Catholic University, Hsin-Chuang City, Taipei County, Taiwan; 2 Division of Mental Health and Addiction Medicine, Institute of Population Health Science, National Health Research Institutes, Zhunan, Miaoli County, Taiwan

**Keywords:** Psychostimulants, METH-induced sensitization, μ-opioid receptor, locomotor activity.

## Abstract

Methamphetamine is a potent addictive stimulant drug that activates certain systems in the brain. It is a member of the amphetamine family, but the effects of methamphetamine are much more potent, longer lasting, and more harmful to the central nervous system. Repeated administration of methamphetamine induces behavioral sensitization, which is considered to be related to compulsive drug-seeking behavior. Although the mechanism responsible for methamphetamine-induced behavioral sensitization remains unclear, it is believed that the mesolimbic dopaminergic system in the central nervous system plays a critical role in the development of behavioral sensitization. Our previous studies indicate that the involvement of the μ-opioid receptor system underlies the development of methamphetamine-induced behavioral sensitization. Understanding the mechanisms of behavioral sensitization that are regulated by the μ-opioid receptor system would be helpful in developing therapeutic programs against methamphetamine addiction. This review briefly discusses the neural circuitry and cellular mechanisms that are known to play a central role in methamphetamine-induced behavioral sensitization and outlines the role of the μ-opioid receptor system in the development of methamphetamine-induced sensitization.

## BACKGROUND

Methamphetamine is a central nervous system stimulant drug that is similar in structure to amphetamine but has a higher potential for abuse, resulting in behavioral changes including sensitization, tolerance, and dependence [1]. It has been suggested that sensitization contributes to both initiation and maintenance of drug addiction [2]. Drug addiction is mediated by multiple brain regions and neurotransmitter systems. The mesolimbic dopaminergic system in the central nerve system is thought to play a critical role in the development of addictive behavior [3, 4] and is strongly modulated by other neurotransmitter systems, including the opioidergic [5], glutamatergic [6], and γ-aminobutyric acid (GABAergic) systems [7]. Many studies have shown that the dopaminergic and opioidergic systems interact in the mesolimbic brain areas. For example, microinjection of[D-Ala2, N-Me-Phe4-Gly5-ol]-enkephalin (DAMGO; a µ-opioid receptor agonist)  caused an increase in the ventral striatal DA and DA-metabolite concentrations in rats [8]. Pharmacological evidence also indicates that acute administration of morphine increases the level of extracellular dopamine in the nucleus accumbens and striatum of mice [9, 10]. Lesions of dopaminergic neurons [11, 12] or neuroleptic blockade [13] of dopamine receptors attenuated opiatereward, as measured by intracranial electrical self-stimulation,conditioned place preference (CPP), and intravenous self-administration. Moreover, our previous studies have shown that blockade of the μ-opioid receptor caused reduced development of methamphetamine-induced sensitization [14-16] and that these results were related to the changes in the levels of dopamine and its metabolites [17], as well as to the binding of dopamine receptors [18]. Therefore, understanding the role the μ-opioid receptor plays in dopaminergic neurotransmission underlying methamphetamine-induced behavioral sensitization would facilitate the discovery of a new therapy program against addiction to psychostimulants. This review briefly outlines the investigation on the role of the μ-opioid receptor in the development of methamphetamine-induced sensitization.

### Development of an Animal Model for Methamphetamine-Induced Behavioral Sensitization

Repeated administration of methamphetamine has been known to produce a progressively enhanced and persistent behavioral response in rodents, a phenomenon called “behavioral sensitization” [14, 19]. Behavioral sensitization has been used as an animal model for studying the development of craving in addicts and psychosis that arises from repeated exposure to psychostimulants [19, 20]. Previous studies have indicated that once sensitization has developed, a challenge dose (low dose) of methamphetamine resulted in behavioral hyperactivity, characterized by increase in locomotor activity and stereotyped behaviors in mice [14, 21]. For example, mice injected with 2.5 mg/kg of methamphetamine once a day for 7 consecutive days showed behavioral hyperactivity after challenge with 0.3125 mg/kg of methamphetamine on day 11 [14]. For the initiation of behavioral sensitization to methamphetamine, mice were usually given a single daily i.p. injection of methamphetamine or saline (control) for 5 to 7 consecutive days. For the evaluation of the expression of behavioral sensitization, the sensitized mice received one injection of methamphetamine (the same dose or lower as for initiation) or saline challenge on day 11 (with 4 abstinent days) or day 21 (with 14 abstinent days). Although the mechanism responsible for methamphetamine-induced behavioral sensitization remains unclear, it is believed that the mesolimbic dopaminergic system in the central nervous system plays a critical role in the development of behavioral sensitization [4, 22, 23]. It has been reported that a challenge dose of methamphetamine (2 mg/kg) markedly increased dopamine turnover (lower dopamine and higher 3, 4-dihydroxyphenylacetic acid (DOPAC) levels, higher ratios of DOPAC over dopamine) in the striatum and nucleus accumbens of the sensitized animals on day 15 of withdrawal from daily treatment with the drug, 6 mg/kg, for 14 days [3]. These findings demonstrate that behavioral sensitization induced by methamphetamine is accompanied by increased central dopaminergic transmission [3, 4, 23].

### Role of µ-Opioid Receptor in Methamphetamine-Induced Sensitization

Topographic overlaps between the μ-opioid receptor and dopamine neurons were found in the central nervous system, suggesting that there are interactions between these two systems [24, 25]. Mu opioid receptor activation has been shown to play a role in psychostimulant-induced gene expression and behavior. For example, pretreatment with the μ-opioid receptor antagonist clocinnamox blocked methamphetamine-induced increases in *zif*268 messenger RNA expression in the dorsal striatum of the rat [26]. Intrastriatal administration of morphine given with a low dose of amphetamine that did not induce stereotypy resulted in a level of stereotypic behavior usually seen with a high dose of amphetamine [27]. Our previous studies found that naltrexone, a long-lasting universal opioid receptor antagonist, could attenuate the induction and expression of methamphetamine-induced behavioral sensitization in NIH Swiss mice [14, 15]. These results show that the expressions of behavioral sensitization were attenuated by pretreatment with 10 or 20 mg/kg of naltrexone either during the induction period or before methamphetamine challenge when they were tested on days 11 and 21 [14]. In addition, radioligand binding revealed that the maximal binding of the μ-opioid receptor, not the binding of delta- and kappa-opioid receptors, was down-regulated on day 8 after 7 daily consecutive administrations of methamphetamine. After cessation of drug treatment, the maximal binding of the μ-opioid receptor gradually and time-dependently returned to normal level on day 11 and up-regulated on day 21. These findings indicate enhanced responsiveness and elevated constitutive activity of the μ-opioid receptor in NIH Swiss mice [15]. Recent evidence also demonstrated that the μ-opioid receptor agonist morphine increased methamphetamine-induced reinforcing behavior and its association with dopamine release in the striatum of mice [28]. In the study, mice were i.p. injected with 0.75 mg/kg methamphetamine and 5 mg/kg morphine alone or in combination to examine the effects of acute (i.e. once), repeated (i.e. once daily for five consecutive days), or challenge administration (i.e. injection of drugs again after 7-day abstinence period) on behaviors including CPP, locomotor activity, and stereotyped behavior, as well as on the levels of dopamine and its metabolites. The results show that the combined administration of methamphetamine and morphine resulted in a greater CPP value than treatment with either methamphetamine or morphine alone. Treatment with methamphetamine resulted in a progressive increase in ambulatory distance, suggesting the development of locomotor sensitization in response to methamphetamine challenge; but there were no significant changes in ambulatory distance for the group of mice injected with morphine during the course of acute, repeated, or challenge administration [28]. Neither the single nor combined regimens of drugs had effects on stereotyped behavior during five consecutive days of repeated administration [28]. In addition, the results revealed that challenge with the combination of methamphetamine and morphine significantly increased striatal dopamine levels compared with challenge with methamphetamine and morphine alone. Challenge with methamphetamine led to a progressive and time-dependent decrease in striatal levels of DOPAC and homovanillic acid (HVA). Neither challenge with morphine nor methamphetamine plus morphine produced significant differences in the DOPAC and HVA levels as compared with saline controls [28]. These findings provide insight into the behavioral and neurochemical basis responsible for the combined abuse liability of methamphetamine and morphine.

### Alternations of Neurochemical Change in Methamphetamine-Induced Sensitization in Wild-Type and µ-Opioid Receptor Knockout Mice

Mu-opioid receptor knockout mice have also shown less behavioral sensitization than wild-type mice after repeated administration of methamphetamine [16]. The effects of behaviors and biochemical factors after blockade or lack of the μ-opioid receptor in methamphetamine sensitization are summarized in Table **1**. Mu-opioid receptor knockout mice injected with 0.625 mg/kg of methamphetamine once a day for 7 consecutive days showed less behavioral hyperactivity as compared with that of the wild-type mice after challenge with 0.625 mg/kg of methamphetamine on day 11 and day 21 [16]. In addition, the levels of dopamine metabolites, DOPAC and HVA, were decreased in the striatum of the wild-type mice — but not in the μ-opioid receptor knockout mice — after 7 consecutive days of treatment followed by 4 days of drug abstinence (on day 11), as measured by *in vivo* microdialysis [17]. These findings suggest that the μ-opioid receptor plays a modulatory role in methamphetamine-induced behavioral sensitization that is related to dopamine metabolism in the mouse striatum. In autoradiographic study, our data showed that there was a decreased binding in the D1 but not the D2 dopamine receptor in the striatum and nucleus accumbens of μ-opioid receptor knockout mice, but not wild-type mice, after repeated administration of methamphetamine [18]. It seems that downregulation of the D1 dopamine receptor is the main factor behind attenuation of behavioral sensitization in μ-opioid receptor knockout mice following administration of methamphetamine. However, another set of experiments showed that the antagonistic effect of haloperidol (a potent D2 dopamine receptor antagonist with a lower affinity for the D1 dopamine receptor) on methamphetamine-induced stereotyped sensitization was more potent in μ-opioid receptor knockout mice than in the wild-type controls [16]. These results reveal that the function of D2 dopamine receptor may turn to be more predominant in μ-opioid receptor knockout mice as compared with that of wild-type controls.

Earlier studies have reported that both selective D1 and D2 dopamine receptor antagonists not only reversed methamphetamine-induced motor effects at each injection but also prevented the development of behavioral sensitization induced by repeated methamphetamine administration [22, 29]. This means that both D1 and D2 dopamine receptor systems are involved in the development of behavioral sensitization to methamphetamine [22, 29]. The D1 and D2 dopamine receptors have opposing actions on the activity of adenylyl cyclase in dopaminergic neurons of the striatum and nucleus accumbens. Activation of the D2 dopamine receptor inhibited adenylyl cyclase activity [30], whereas activation of the D1 dopamine receptor increased adenylyl cyclase activity [31]. These actions resulted in a decrease in the level of cAMP and protein kinase A signal, which caused a decrease in the phosphorylation of DARPP-32 (dopamine- and cAMP-regulated phosphoprotein of Mr 32 kDa)-dependent signaling pathway [32, 33]. Moreover, through the cAMP/PKA, activation of D2 dopamine receptor inactivated phosphorylation of DARPP-32 through increasing calcium levels in the striatum and nucleus accumbens [32]. This means that activation of the D2 dopamine receptor can inactivate phosphorylation of DARPP-32 through cAMP/PKA or Ca^2+^/calcineurin. It has also been reported that phosphorylation of DARPP-32 may be involved in acute administration of cocaine or amphetamine that leads to an increase in locomotor activity [34]. These responses were severely attenuated in DARPP-32-deficient mice [34, 35]. These data reveal that phosphorylation of DARPP-32 may play an important role in the mechanism of behavioral sensitization induced by psychostimulants such as methamphetamine in animals.

## SUMMARY

The findings summarily indicate that the μ-opioid receptor plays an important role in modulating the development of methamphetamine-induced behavioral sensitization through dopaminergic neurotransmission. Understanding the regulatory mechanism of the μ-opioid receptor on cellular signaling pathways and nuclear gene expression of dopaminergic system during the development of methamphetamine-induced behavioral sensitization would help us to develop therapeutic programs against drug addiction. Thus, further studies are necessary.

## Figures and Tables

**Table 1.** Effect of Blockade or Lack of µ-Opioid Receptor on Behaviors and Neurochemical Factors in Methamphetamine-Induced Sensitization

	Effect	Reference
METH-induced behaviors		
Locomotor activity	↓	Chiu *et al.*, 2005; Shen *et al*., 2010
Stereotypy	↓	Shen *et al.*, 2010
Dopaminergic system affected by METH		
Levels of dopamine metabolites (DOPAC and HVA)	↑	Lan *et al.*, 2008
Receptor binding	D1↓; D2↑	Tien *et al.*, 2007
Others		
[^3^H]-DAMGO binding	↑	Chiu *et al.*, 2006
DAMGO-stimulated [^35^S]GTP_γ_S binding	↓	Chiu *et al.*, 2006
Preprodynorphin mRNAs expression	↓	Horner and Keefe, 2006
Preproenkephalin mRNAs expression	↓	Tien *et al.*, 2007
*zif*268 mRNAs expression	↓	Horner and Keefe, 2006

↑ Increase

↓ Decrease.

DOPAC: 3, 4-dihydroxyphenylacetic acid, HVA: homovanillic acid.

DAMGO: [D-Ala^2^, N-MePhe^4^, Gly-ol]-enkephalin (µ-opioid receptor agonist).
